# Visual–spatial abilities enhancement and spatial anatomy learning: A systematic review

**DOI:** 10.1111/medu.70022

**Published:** 2025-08-19

**Authors:** Lara Nokovitch, Houssein El Hajj, Sophie Deneuve, Vincent De Andrade, Rémi Gagnayre, Aurore Margat

**Affiliations:** ^1^ Maxillo‐Facial Surgery Department Beaujon University Hospital Clichy France; ^2^ Anatomy teaching department Paris Cité University Paris France; ^3^ Education and Health Promotion Laboratory (LEPS), (UR3412), UFR SMBH Sorbonne Paris‐Nord University Bobigny France; ^4^ Gynecologic Oncology Surgery Department Institut Gustave Roussy Villejuif France; ^5^ ENT and Head and Neck Surgery Department Rouen University Hospital Rouen France

## Abstract

**Introduction:**

Student's visual–spatial abilities appear to be an important predictor of learning time, performance and knowledge in anatomy. The objective of this systematic review was to assess the relationship between spatial abilities enhancement and spatial learning of anatomy.

**Materials and Methods:**

A systematic literature search of databases (MEDLINE, Embase, ERIC, Scopus and Web of Science) was undertaken using relevant keywords. The search included citations published from inception to December 31st, 2024. The transferability of spatial abilities enhancement to spatial learning of anatomy was assessed using a 2 × 2 classification of spatial skills, in which each visual–spatial task was coded in terms of both intrinsic‐extrinsic and static‐dynamic dimensions. A qualitative analysis of the results was performed.

**Results:**

The search yielded 2135 results, which narrowed down to 8 articles after application of exclusion criteria. Significant relationships between spatial abilities and the spatial learning of anatomy were observed in five studies. Among studies reporting a positive correlation between spatial abilities and spatial learning of anatomy, within‐cell transfer was observed for four studies, and across‐cell transfer for one study.

**Conclusions:**

Enhanced spatial abilities seem to transfer mainly to similar tasks related to spatial learning of anatomy. However, the spatial abilities tests used to evaluate spatial abilities were all intrinsic × dynamic. A future study using different spatial abilities tests could evaluate if spatial abilities engaging different dimensions of spatial cognition could be related to different spatial anatomy tasks.

## Abstract 2

Nokovitch et al. detail how and when enhancing spatial abilities transfers to anatomy learning while raising critical insight into limitations of the way we measure spatial skill.

## 1 INTRODUCTION

### 1.1 The process and difficulties of learning anatomy

Anatomy learning requires the construction of knowledge using visual models, higher anatomy achievement being reliant on a firm understanding of 3 dimension (3D) relationships between anatomical structures.^1^, ^2^ According to Mayer et al, the cognitive process of learning from visual models involves the selection, organization and integration of images into a coherent mental representation, and is commonly referred to as visual–spatial thinking.^3^, ^4^, ^5^, ^6^, ^7^ The development of visual–spatial skills is a prerequisite for the development of students' reasoning skills, and the understanding of static and dynamic relationships between anatomical structures.^8^ The difficulties experienced by medical students in anatomy are largely dominated by the feeling that they cannot visualize 3D structures and cannot orient them in the material being studied.^9^ Some topics also seem to be more problematic than others (pelvis, neuroanatomy, perineum, omentum and linings of the body cavities), probably owing to the complicated nature and restricted access to certain regions, and the inability to conceptualize these areas.^9^ This inability to reconstruct or visualize 3D representations from 2 dimension (2D) images may be due to inadequate guidance regarding how to conceptualize 3D structures, especially for students with low visual–spatial skills.^9^ Laboratory classes and other learning activities involving 3D information (3D models, virtual‐reality, cross‐sections, etc.) can help students to engage in visual–spatial thinking and the associated learning of anatomical technical terminology.^10^ However, unless students are actively encouraged to engage, learning may be passive and difficult to transfer to a clinical situation.^10^ This frequent lack of clinical integration impedes the “near” and “far” transfer of learning.^11^ Learners also struggle to cope with the large amount of knowledge, which is a pre‐requisite to successful deep understanding of concepts and transfer of learning.^11^

Recent literature also suggests that haptics could be a useful cognitive resource for anatomy learning, mental representations afforded by tactile sensory inputs leading to more meaningful learning, as better cognitive schema may be built from representations obtained from diverse multimodal sensory inputs.^12^ Whereas visuo‐spatial abilities are predominantly driven by vision, haptic abilities consist of tactile interactions with real‐world objects, transforming somatic information into mental representations.^12^ To explore the relationship between haptic and visuo‐spatial abilities, Langlois et al asked medical graduates to draw different objects from blind haptic perception, and used vision‐based tests.^13^ Spatial abilities assessed by vision‐based tests were correlated with a drawing score based on haptic perception of objects.^13^ Conversely, Sveistrup et al measured visual and haptic behaviours during spatial tasks, and showed that haptic abilities were unrelated to visuo‐spatial abilities.^14^ However, subjects with low visuo‐spatial abilities tended to use haptic strategies proportionally more than subjects with high visuo‐spatial abilities, suggesting that multimodal sensory inputs could contribute to problem‐solving behaviours when visual–spatial abilities are challenged.^14^

### 1.2 Measurement of visual–spatial abilities

Prior attempts at defining and classifying spatial abilities have mostly followed a psychometric approach.^15^ Several categories of spatial abilities have been identified,^16^, ^17^, ^18^ and although there is no clear consensus regarding the definition and subcomponents of spatial ability, agreement seems to be the strongest for the existence of visual–spatial abilities.^15^ The ability to visualize a 2D or 3D object, and then transform a mental image of the object, has been assessed quantitatively in the field of cognitive psychology using context‐specific tests.^2^ Among them, the Vandenberg Mental Rotation Test (VMRT), previously validated by Vandenberg and Kuse (1978), has been widely used in the assessment of visual–spatial abilities.^19^ It consists of a standard set of 24 items with within each item, a 3D figure presented as a 2D drawing with four possible rotated versions. Subjects have to mentally rotate the figure in order to identify the correct items.^19^ A positive association has been shown between the VMRT and anatomy learning.^2^ However, a testing effect has been previously reported in the case of repeated administration.^20^, ^21^, ^22^ Other psychometric tests, such as the Purdue Spatial Visualization Test of Rotations (PSVT:R) validated by Carter, LaRussa and Bodner (1987), can also be used to assess mental rotation ability.^23^ In a similar process, it measures the ability of a subject to recognize the rotation of an object, before performing the same mental rotation on a second object.^23^ Nevertheless, the association between these tests and anatomy learning is less clear than for the VMRT.^23^

To provide a more precise description of visual–spatial skills and their corresponding tests, Uttal et al proposed a 2 × 2 classification of spatial skills, in which each visual–spatial task is coded in terms of both intrinsic‐extrinsic and static‐dynamic dimensions.^15^ Spatial tasks that involve defining an object can be coded as intrinsic, whereas spatial tasks that require the participant to determine relations among objects in a group can be coded as extrinsic.^15^ Likewise, spatial tasks in which the main object remains stationary can be coded as static, whereas spatial tasks in which the main object moves, either physically or in the mind of the participant, can be coded as dynamic.^15^

### 1.3 Visual–spatial abilities enhancement and spatial learning of anatomy

Student's visual–spatial abilities appear to be an important predictor of learning time, performance and knowledge in anatomy,^1^, ^21^, ^24^, ^25^ the acquisition of anatomical knowledge depends on student's ability to construct visual–spatial 3D representations out of 2D images and to mentally manipulate these representations.^1^, ^25^, ^26^, ^27^, ^28^ Assuming that anatomy knowledge includes both spatial and non‐spatial components, Langlois et al explored the relationship between spatial abilities test and anatomy knowledge assessment in a systematic review. Significant relationships were observed when using practical examination, 3D synthesis from 2D views, drawing of views and cross‐sections; whereas non‐significant relationships were found when using essays and non‐spatial multiple‐choice questions, providing evidence for spatial and non‐spatial methods of anatomy knowledge assessment.^2^

Multiple studies tried to confirm the relationships between spatial abilities and anatomy performance with mixed conclusions, ranging from no existing correlation^29^, ^30^, ^31^ to positive correlations of various magnitudes.^23^, ^25^, ^32^ In an attempt to objectively summarize the effects of spatial ability on anatomy assessment performance, Roach et al in a comprehensive meta‐analysis explored the relationship between spatial abilities and anatomy performance scores.^1^ Spatial abilities were assessed using a VMRT. Across 15 studies and 1245 participants, they concluded that spatial ability was weakly associated with anatomy performance.^1^ In agreement with Langlois et al previous results,^2^ performance on spatial and relationship‐based assessments was correlated with spatial ability, while performance on assessments utilizing non‐spatial multiple‐choice items was not correlated with spatial ability.^1^

Spatial abilities also appear to be responsive to training and experience.^15^ Langlois et al in a systematic review focusing on training strategies to improve spatial thinking, showed that anatomy learning and mental rotation training could improve students' spatial abilities.^33^ Other educational materials, such as video games, have also been shown to improve visual–spatial skills.^15^ However, Langlois et al pointed out that “future studies will need to validate if this enhancement in spatial abilities is related to spatial learning of anatomy”.^33^ To determine the size of spatial training effects, as well as whether any such training effects are durable and can transfer to new tasks, Uttal et al meta‐analysed 217 studies investigating the magnitude, moderators, durability and generalizability of training on spatial skills.^15^ Training effects on spatial abilities appear to be stable and not affected by delays between training and post‐testing.^15^ Training also seems to transfer to other spatial tasks that were not directly trained.^15^

### 1.4 Objectives and aims

The main objective of this systematic review was to assess the relationship between spatial abilities enhancement and spatial learning of anatomy.

## 2 MATERIALS AND METHODS

### 2.1 Databases and search strategy

A comprehensive search was designed and executed by a health sciences librarian in MEDLINE (US National Library of Medicine, Bethesda MD), Embase (Elsevier, Amsterdam, The Netherlands), ERIC (Institute of Education Sciences, Washington, DC), Scopus™ (Elsever B.V., Amsterdam, The Netherlands) and Web of Science from inception to December 31st, 2024 with no filters and no language restriction. The search included citations published from inception to December 31st, 2024. Search strategies were listed in Supplementary Material 1.

### 2.2 Screening of citations and full‐text articles

**FIGURE 1:**
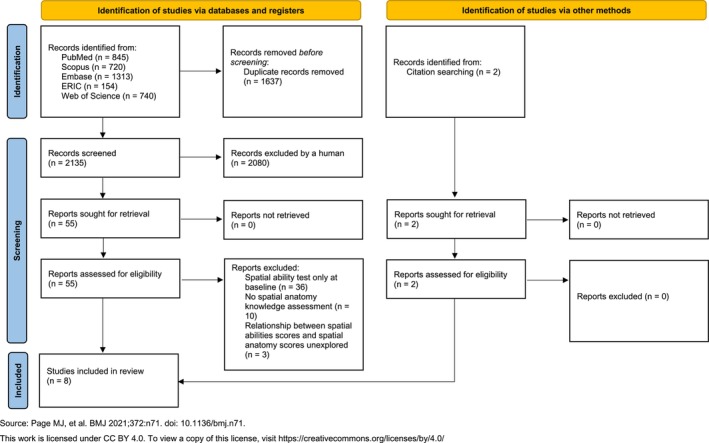
**PRISMA flow‐chart of the study** (McKenzie JE, Bossuyt PM, Boutron I, Hoffmann TC, Mulrow CD, et al The PRISMA 2020 statement: an updated guideline for reporting systematic reviews. BMJ 2021;372:n71. doi: 10.1136/bmj.n71). [Color figure can be viewed at wileyonlinelibrary.com]

Citations (title and abstract) were reviewed by two independent reviewers, conflicts between reviewers were discussed until a consensus was reached, and a third reviewer was involved if needed. References lists of articles identified for inclusion were screened for additional citations. Citations related to conference abstracts, books, books sections and theses were excluded. Citations were included if: 1) they involved the field of anatomy education, 2) a valid and reliable spatial ability test was performed before and after a training program known to increase spatial skills (ex: anatomy course, mental rotation training, video games),^33^ 3) there was an enhancement of spatial ability scores between the pre‐ and post‐test, 4) anatomy knowledge was assessed by spatial methods, 5) the relationship between spatial abilities and anatomy scores using spatial methods was evaluated. All five of these criteria had to be satisfied for an article to be included. Rayyan software was used to assist in the database screening for inclusion criteria. Full‐text articles were reviewed by two independent reviewers. Conflicts between reviewers were discussed until a consensus was reached, and a third reviewer was involved if needed. The Preferred Reporting Items for Systematic reviews and Meta‐Analysis (PRISMA) flow‐chart^34^ was utilized for the report of the findings (Figure 1).

### 2.3 Review of selected articles

The reviewers reread the included studies and independently extracted relevant information onto a predetermined data form created with input from all the authors. The following data were extracted from eligible articles: study type, sample size, information related to PICO framework – Population (number and type of participants, country, sex and age), Intervention (training course known to increase spatial abilities), Comparator (spatial anatomy knowledge assessment method) and Outcomes (reported relationship between spatial abilities enhancement and anatomy scores using spatial anatomy assessment methods).^35^ Independent data extraction was performed by the authors. All the authors reviewed the data collected and came to a consensus on the prominent trends to be featured.

Descriptive analysis was used to report the relationships between spatial abilities enhancement and spatial learning of anatomy. Spatial learning of anatomy was assessed by spatial methods of anatomy knowledge assessment. Spatial methods of anatomy knowledge assessment were previously defined by Langlois et al as practical examination, 3D synthesis from 2D views, drawing of views and cross‐sections.^2^ Cohen's *d* effect sizes were interpreted according to Cohen's convention (small = 0.20, medium = 0.50, large = 0.80).^36^

To assess if spatial abilities enhancement could transfer to spatial learning of anatomy, spatial skills measured by spatial ability tests and spatial methods of anatomy knowledge assessment were both coded into cells of Uttal et al 2 × 2 framework.^15^ Spatial skills consisting of perceiving objects, paths or spatial configurations amid a distracting background, were described as **intrinsic and static.** ^15^ Spatial skills consisting of piecing together objects into more complex configurations, visualizing and mentally transforming objects, often from 2D to 3D, or vice‐versa, and rotation 2D or 3D objects were described as **intrinsic and dynamic.** ^15^ Spatial skills consisting of understanding abstract spatial principles, such as horizontal invariance or verticality, were described as **extrinsic and static.** ^15^ Spatial skills consisting of visualizing an environment in its entirety from a different position, were described as **extrinsic and dynamic.** ^15^ Uttal et al 2 × 2 framework was used to define two levels of transfer. Within‐cell transfer was coded when spatial skills measured by spatial ability tests and spatial methods of anatomy knowledge assessment were not the same, but both in the same cell of the 2 × 2 framework. Across‐cell transfer was coded when spatial skills measured by spatial ability tests and spatial methods of anatomy knowledge assessment were in different cells of the 2 × 2 framework.

## 3 RESULTS

The literature search yielded 3772 citations plus 2 additional citations from references lists of studies identified for inclusion. After the duplicates were removed, 2135 citations were identified, of which 2080 were deemed ineligible, and 56 were considered for full‐text review. Of the 55 articles, 8 were considered eligible and were included in the systematic review, and 49 were excluded for the following reasons: 1) spatial ability test only at baseline (n = 36), 2) no spatial anatomy knowledge assessment (n = 10), 3) relationship between spatial abilities and spatial anatomy scores unexplored (n = 3) (appendix 2).

### 3.1 Characteristics of the included studies

The characteristics of the included studies are described in Table 1. The quality evaluation of the included studies is reported in Appendix 3. Among the eight studies included in the systematic review, the types of study designs were randomized experiments (n = 2), intervention and control group with pre‐test and post‐test design (n = 3) and intervention group with only post‐test design (n = 3). Randomized experiments were of a prospective nature, and participants were randomly assigned to two or more groups using a known randomization technique. Groups were assigned concurrently, after which the intervention was applied, then measured and compared. Intervention group only with post‐test design involved only one group where a pretest or baseline measure was taken and used as a control. Afterwards, an intervention was implemented, and a post‐test measure was taken for comparison. Intervention and control groups with pre‐test and post‐test design involved two groups where the intervention was implemented in one group and compared with a second group without the intervention, based on a post‐test measure from both groups.

**TABLE 1:** Characteristics of the included studies.

Article, year, country	Study design and number of participants (n)	Population (type of participants, sex, age)	Intervention	Type of spatial abilities test	Spatial abilities test score improvement	Spatial anatomy assessment methods	Relationship between spatial abilities test score and spatial anatomy knowledge score
Provo et al., 2002 USA	Randomized experiment (n = 188)	First‐year veterinary students (male = 53, female = 135), mean age = 23.9	**• Experimental group**: dissection on a canine cadaver + cross‐section of a canine head. **• Control groups**: dissection on a canine cadaver + radiographs of the dissected region.	PSVT ***Intrinsic × Dynamic***	NS	Drawing of views ***Intrinsic × Dynamic*** (drawing structures in correct relation to one another) Cross‐sections ***Intrinsic × Static*** (labelling structures in a given cross‐section) ***Extrinsic × Static*** (determining at what level the cross‐section was taken) Practical examination ***Intrinsic × Static*** (recognizing structures within a cadaver)	Positive correlations between spatial abilities test scores and drawing of views (p < 0.05)
Hontoir et al, 2022 Belgium	Randomized experiment (n = 83)	Second‐year veterinary students (male = 19, female = 64), mean age = 20.6	Equine radio‐anatomy practical activity. **• Group A**: digitalized radiographs. **• Group B**: digitalized radiographs + corresponding set of equine bones. **• Group C**: conventional radiographs + corresponding set of equine bones.	VMRT ***Intrinsic × Dynamic***	Statistically significant (p < 0.01)	3D synthesis from 2D views ***Intrinsic × Dynamic***	NS
Hegarty et al., 2009 USA	Intervention group only with post‐test design (n = 79)	First‐year dentistry students (male = 52, female = 27), mean age = unspecified	First‐year dental curriculum including anatomy content.	Spatial anatomy tasks ***Intrinsic × Dynamic***	Statistically significant (p < 0.01)	Cross‐sections ***Intrinsic × Dynamic*** (inferring the cross‐section of a novel 3D object based on a specific slicing plane)	Positive correlations between spatial abilities test scores and cross‐sections (p < 0.05)
Lufler et al., 2011 USA	Intervention group only with post‐test design (n = 352)	First‐year medical students (male = 166, female = 186), mean age = unspecified	Medical gross anatomy course.	VMRT ***Intrinsic × Dynamic***	Statistically significant (p < 0.0001)	Practical examination ***Intrinsic × Static*** (recognizing structures on cadavers and articulated skeletons) ***Intrinsic × Dynamic*** (recognizing structures on a disarticulated skeleton and on removed organs randomly placed)	Positive trend between spatial abilities test scores and practical examination (p < 0.05)
Cui et al., 2017 USA and Canada	Intervention and control group with pre‐test and post‐test design (n = 39)	First‐year medical students (male = 17, female = 22), mean age = unspecified	**3D group**: stereoscopic model of head and neck anatomy. **2D group**: flat screen session of head and neck anatomy.	VMRT ***Intrinsic × Dynamic***	Statistically significant (p < 0.05)	3D synthesis from 2D views ***Intrinsic × Dynamic***	Positive correlations between spatial abilities test scores and anatomy knowledge scores using a combination of spatial and non‐spatial anatomy assessment methods (p < 0.05)
Guimaraes et al., 2018 Portugal	Intervention and control group with pre‐test and post‐test design (n = 611)	Medical students (male = 212, female = 399), mean age = 21.2	Computer‐assisted learning sessions. **• Musculoskeletal anatomy (MA) group**: 15 musculoskeletal anatomy training sessions. **• Cardiovascular anatomy (CA) group**: 12 cardiovascular anatomy training sessions. **• Musculoskeletal + Cardiovascular anatomy (MA + CA) group**: 15 musculoskeletal anatomy training sessions + 12 cardiovascular anatomy training sessions.	VMRT ***Intrinsic × Dynamic***	Statistically significant (p < 0.001)	Practical examination ***Intrinsic × Static*** (recognizing structures within a fixed anatomical region)	Positive correlations between spatial abilities test scores and digital‐based practical examination (p < 0.05)
Yousuf et al., 2023 Jordan	Intervention and control group with pre‐test and post‐test design (n = 89)	Second and fifth‐year medical students (male = 36, female = 53), mean age = unspecified	**• Group A**: general anatomy course through online videos. **• Group B**: face‐to‐face general anatomy course.	VMRT ***Intrinsic × Dynamic***	NS	Practical examination ***Intrinsic × Static*** (recognizing structures within a fixed anatomical region)	NS
Harmon et al., 2022 USA	Intervention group only with post‐test design (n = 49)	First year physical therapy students (male = 16, female = 33), mean age = 25.8	Gross neuromuscular anatomy course (lectures, case‐based learning, prosection).	VMRT ***Intrinsic × Dynamic***	Statistically significant (p < 0.0001)	Practical examination ***Intrinsic × Static*** (recognizing structures within a fixed anatomical region)	NS

NS: not statistically significant, NA: not available, VMRT: Vandenberg Mental Rotation Test.

Interventions in the included studies were heterogeneous and included dissection in one study [Provo et al, 2002^23^], prosection in one study [Harmon et al, 2022^37^], 2D imaging in two studies [Provo et al, 2002^23^; Hontoir et al, 2022^35^], 3D anatomical models in one study [Hontoir et al, 2022^35^], computer‐assisted learning sessions in two studies [Cui et al, 2017^38^; Guimaraes et al, 2018^39^; Yousuf et al, 2023^40^], virtual reality in one study [Cui et al, 2017^38^], lectures in one study [Harmon et al, 2022^37^] and case‐based learning in one study [Harmon et al, 2022^37^]. In two studies, the educational resources used to teach anatomy were not specified [Hegarty et al, 2009^32^; Lufler et al, 2011^26^]. Mixed‐learning activities were used in only three studies [Provo et al, 2002^23^; Hontoir et al, 2022^35^; Harmon et al, 2022^37^] (Table 1).

A summary of participants' demographics is provided in Table 1. Several types of study participants were considered among the eight studies: medical students (n = 1091), veterinary students (n = 271), dentistry students (n = 79) and physical therapy students (n = 49). The countries in which the studies were conducted included: Belgium (n = 1), Canada (n = 1), the United States (n = 5), Portugal (n = 1) and Jordan (n = 1). A total of 1490 participants were identified. Among them, 919 (61.6%) were females and 571 (38.4%) were males. Participants' mean age was 22.8 years for 931 participants, and not specified for 559 participants.

Spatial abilities tests and spatial methods of anatomy knowledge assessment were identified in the included studies and are presented in Table 1. Spatial abilities tests were all coded as intrinsic × dynamic according to Uttal et al 2 × 2 framework (Table 1). Spatial methods of anatomy knowledge assessment included drawing of views in one study [Provo et al, 2002^23^], cross‐sections in two studies [Provo et al, 2002^23^; Hegarty et al, 2009^32^], practical examinations in five studies [Provo et al, 2002^23^; Lufler et al, 2011^26^; Guimaraes et al, 2018^39^; Yousuf et al, 2023^40^; Harmon et al, 2022^37^] and 3D synthesis from 2D views in two studies [Hontoir et al, 2022^35^; Cui et al, 2017^38^]. Spatial methods were later coded according to Uttal et al 2 × 2 framework as intrinsic × dynamic in five studies [Provo et al, 2002^23^; Hontoir et al, 2022^35^; Hegarty et al, 2009^32^; Lufler et al, 2011^26^; Cui et al, 2017^38^], intrinsic × static in five studies [Provo et al, 2002^23^; Lufler et al, 2009^26^; Guimaraes et al, 2018^39^; Yousuf et al, 2023^40^; Harmon et al, 2022^37^] and extrinsic × static in one study [Provo et al, 2002^23^]. Some spatial anatomy assessment methods often engaged multiple dimensions of spatial cognition as defined by Uttal et al. 2 × 2 framework (Table 1). Depending on the task demands and the context, they could involve both intrinsic and extrinsic spatial relations, as well as static and dynamic processing. As such, these methods did not fit into a single category and covered more than one quadrant of the framework.

### 3.2 Descriptive analysis

#### 3.2.1 Spatial abilities enhancement

Spatial abilities enhancement was statistically significant in six out of eight studies [Hontoir et al, 2002^35^; Hegarty et al, 2009^32^; Lufler et al, 2011^26^; Cui et al, 2017^38^; Guimaraes et al, 2018^39^; Harmon et al, 2022^37^]. In two remaining studies, the p‐value was not statistically significant, but spatial ability scores tended to improve between the pre‐ and post‐test [Provo et al, 2002^23^; Yousuf et al, 2023^40^]. When statistically significant, the improvement of spatial abilities test scores was secondary to an intervention using 2D imaging in one study [Hontoir et al, 2022^35^], computer‐assisted learning sessions in two studies [Cui et al, 2017^38^; Guimaraes et al, 2018^39^], virtual reality in one study [Cui et al, 2017^38^], mixed‐learning activities including 2D imaging and 3D anatomical models in one study [Hontoir et al, 2022^35^]; and lectures, case‐based learning and prosection in one study [Harmon et al, 2022^37^].

#### 3.2.2 Spatial learning of anatomy

Out of the eight studies included, five showed significant relationships between spatial abilities and anatomy knowledge assessment scores using spatial methods such as cross‐sections [Hegarty et al, 2009^32^], drawing of views [Provo et al, 2002^23^], 3D synthesis from 2D views [Cui et al, 2017^38^] and practical examination [Lufler et al 2011^26^; Guimaraes et al 2018^39^], whereas no significant relationships were found in three studies using 3D synthesis from 2D views [Hontoir et al, 2022^35^], and practical examination [Yousuf et al, 2023^40^; Harmon et al, 2022^37^](Table 1).

Positive correlations between spatial abilities and spatial learning of anatomy were found to be **weak** (correlation coefficients between 0.10 and 0.20) in two studies [Provo et al, 2002^23^; Guimaraes et al, 2018^39^], and **moderate** (correlation coefficients between 0.21 and 0.50) in two studies [Hegarty et al, 2009^32^; Guimaraes et al, 2018^39^]. The effect of high visual–spatial abilities on practical examination was noted by Lufler et al,^26^ who showed that students who scored in the highest quartile on the VMRT had a significantly higher mean practical examination score than students who scored in the lowest quartile on the VMRT (p = 0.03) (Table 1).

The transferability of spatial abilities enhancement to spatial learning of anatomy was assessed using Uttal et al 2 × 2 framework. Among studies reporting significant relationships between spatial abilities and spatial learning of anatomy, within cell‐transfer was observed for four studies [Provo et al, 2022^23^; Hegarty et al, 2009^32^; Lufler et al, 2011^26^; Cui et al, 2017^38^], and across‐cell transfer for two studies [Lufler et al, 2011^26^; Guimaraes et al, 2018^39^](Table 1). It is to note that in Lufler et al study,^26^ spatial anatomy scores for practical examination included the results of both intrinsic × static and intrinsic × dynamic spatial anatomy assessment methods without distinction.

A significant relationship between spatial abilities enhancement and anatomy scores was observed after heterogeneous interventions including dissections in one study [Provo et al, 2002^23^], 2D imaging in one study [Provo et al, 2002^23^], computer‐assisted learning in two studies [Cui et al, 2017^38^; Guimaraes et al, 2018^39^], virtual reality in one study [Cui et al, 2017^38^] and undefined anatomy interventions in two studies [Hegarty et al, 2009^32^; Lufler et al, 2011^26^].

## 4 DISCUSSION

### 4.1 Spatial abilities enhancement and spatial learning of anatomy

The present study used a systematic review to assess the relationship between spatial abilities enhancement and spatial learning of anatomy. All the participants of the included articles presented increased spatial abilities after they received an intervention in anatomy. When statistically significant, the improvement of spatial abilities test scores was secondary to an intervention using 2D imaging, computer‐assisted learning sessions, virtual reality and mixed‐learning activities. These findings are an incremental contribution to Langlois et al systematic review on spatial anatomy training in anatomy education.^33^ Indeed, he concluded that heterogeneous interventions in anatomy increased students' spatial abilities, without categorizing the learning activities used to train spatial abilities.^33^ A future study could evaluate if mixed‐learning activities combining multiple pedagogical resources, found to improve spatial abilities, could be more effective to train students' spatial abilities.

Among the included studies, significant relationships between spatial abilities and the spatial learning of anatomy were observed in five studies. Positive correlations between spatial abilities and spatial learning of anatomy were found to be weak to moderate. These results are similar to those of Roach et al, who reported in their meta‐analysis a weak pooled correlation coefficient (r_pooled_ = 0.240) between spatial abilities and learning of anatomy.^1^ However, in Roach et al.'s meta‐analysis, no distinction was made between spatial and non‐spatial learning of anatomy, and a spatial ability pre‐ and post‐test was performed in only three studies.

In the literature, improvement of spatial abilities in a given field was not found to transfer necessarily to another.^41^ Furthermore, the translation of improved spatial abilities toward better knowledge in the STEM fields has not been established.^33^ In our study, the method used to assess the transferability of spatial abilities enhancement to spatial learning of anatomy was based on qualitative definitions of intrinsic, extrinsic, static and dynamic spatial abilities.^15^ Spatial anatomy assessment methods engaged sometimes multiple dimensions of spatial cognition as defined by Uttal et al 2 × 2 framework.^15^ Depending on the task demands and the context, they could involve both intrinsic and extrinsic spatial relations, as well as static and dynamic processing. As such, these methods could not fit into a single category, and covered more than one quadrant of the framework, pointing out the limits of Langlois et al definitions of spatial methods,^33^ and the difficulty to assess the spatial and non‐spatial character of assessment methods. Among studies reporting a positive correlation between spatial abilities and spatial learning of anatomy, within‐cell transfer was observed in most studies, suggesting evidence for transfer between spatial abilities enhancement and tasks related to spatial learning of anatomy requiring similar skills or representations. Across‐cell transfer was also observed in one study, suggesting the existence of transfer between spatial abilities enhancement and tasks related to spatial learning of anatomy that might require different skills or representations. These results might indicate that enhanced spatial abilities transfer mainly to similar tasks related to spatial learning of anatomy. Unfortunately, the degree or range of transfer could not be evaluated, as we were unable to calculate the effect size for within‐cell and across‐cell transfer due to the small number of articles included. Moreover, the spatial abilities tests used to evaluate spatial abilities in the included articles were all intrinsic × dynamic. A future study using different spatial abilities tests could evaluate if spatial abilities engaging different dimensions of spatial cognition could be related to different spatial anatomy tasks.

### 4.2 Implications for anatomy instruction

The choice of the intervention when spatial abilities are involved is contrasted in the literature,^38^, ^42^, ^43^ and advancing technology in medical education has led to increasing research on the effectiveness of various learning activities in anatomy.^44^ The role of stereopsis, referring to the experience of spatial depth based on the brain's comparison of synchronous and overlapping information provided by binocular vision,^42^ and haptics, referring to tactile interactions with real‐world objects transforming somatic information into mental representations, must also be taken into account when learning spatial anatomy.^12^ In our study, significant relationships between spatial abilities and anatomy scores were observed after various interventions including dissections, 2D imaging, computer‐assisted learning and virtual reality. Traditional dissection enables interactive visio‐tactile information in a way that no other learning activity can adequately replicate to synthetize meaning and comprehension.^45^ Moreover, in a comparative study, cadaveric dissection appears to be superior to virtual and mixed reality technologies to learn anatomy, as it enables true stereopsis and haptic perception.^4^ However, dissection is inadequate as a sole method of learning, as it is largely deficient in demonstrating discrete structures such as microsurgical vasculature, functional anatomy and more, therefore necessitating the use of adjunctive modalities.^45^ Medical imaging in anatomy education provides in vivo visualization of anatomical structure and physiology, as well as insight into pathological processes.^46^ It is considered a valuable addition to dissection‐based instruction, as it can promote better understanding of anatomical spatial relationships.^46^ Inspecting medical images also introduces a level of abstraction compared to cadaveric dissection.^46^ Nevertheless, it has important limitations as a stand‐alone approach, numerous anatomical structures with complex courses being difficult to view adequately with current imaging modalities.^46^ In previous literature reviews and recent meta‐analysis, computer‐assisted learning yielded better results over both traditional lectures, 3D anatomical models and prosection.^44^ However, the loss of spatial information caused by digital mediation of 2D and 3D computer models makes it harder to interpret spatially complex objects or contexts.^42^ Hacket et al also showed that 3D images without visual depth cue were no better than printed images, and inferior to autostereoscopic printed images.^47^ To implement the visual depth cue of stereopsis, the dynamic movement of computerized 3D anatomical models seems to be of assistance to low‐spatial abilities subjects performing spatial anatomical tasks.^43^ Virtual reality allows to implement stereopsis digitally. But although immersion in a virtual reality environment can lead to a heightened sense of presence, object manipulation in such an environment is not intuitive and may add to the users' cognitive load.^42^ Finally, virtual remains inferior to physical models, as true stereopsis remains critical in learning anatomy.^4^

The role of image and cognitive load in anatomy learning is central for an appropriate use of multimedia principles, generally including pictures, images and visualizations that will positively influence student's attention and learning, and will benefit to both low and high‐spatial‐ability students.^5^ According to Mayer's cognitive theory of multimedia learning, multimedia learning occurs when students build mental representations from words and pictures that are presented to them.^6^ In multimedia learning, the student engages in three important cognitive processes: 1) selecting: applied to incoming verbal information to yield a text base and to incoming visual information to yield an image base; 2) organizing: applied to the text base to create a verbally‐based model of the system, and to the image base to create a visually‐based model of the system; 3) integrating: when the student builds connections between corresponding events in the verbally‐based model and the visually‐based model.^7^ A multimedia effect is shown when students learn more deeply from words and pictures than from words alone, in both book‐based and computer‐based environments.^6^

Both cognitive load theory and cognitive theory of multimedia learning postulate that the quality of multimedia design heavily influences anatomy learning.^48^ Based upon these learning theories, it is possible to design relevant instructional strategies in anatomy education to facilitate learning for students with different spatial abilities.^1^ Thus, Mayer provided us with five principles of multimedia design to help students understand scientific information using a multimedia learning environment.^7^ The multiple representation principle, stating that it is better to present an explanation in words and pictures than solely in words.^7^ The contiguity principle, arguing that students understand better an explanation when corresponding words and pictures are presented contiguously.^7^ The split‐attention principle, saying that words should be presented auditorily rather than visually.^7^ The individual differences principle, stating that contiguity effects and split‐attention effects depend on individual differences.^7^ And the coherence principle, explaining that students learn better from a coherent summary using few rather than many extraneous words and pictures.^7^ Likewise, the theoretical cognitive process of visualization of Mnguni et al deriving from Mayer's cognitive theory of multimedia learning, suggests that relevant instructional strategies should focus on the internalization, conceptualization and externalization of visual models.^3^

Memorization, understanding and visualization are perceived by students as successful approaches in learning anatomy.^49^ Pandey et al also distinguished a deep and surface approach to learning anatomy, academic performances and quality learning being correlated with a deep approach to learning.^49^ In Pandey et al study, memorization strategies were associated with surface approaches, whereas visualization and understanding were associated with deep approaches to learning anatomy.^49^ Therefore, carefully structuring the curriculum to promote the development of conceptual frameworks should be a priority, especially for students with low spatial abilities.^49^ Instructional approaches utilizing visual cueing in anatomy might also help to improve students' interpretation of visual content, especially for those with low visual–spatial skills.^50^

### 4.3 Study limitations

The search strategy was not revised by a second professional librarian. The number of articles assessing the relationship between spatial abilities enhancement and the spatial learning of anatomy is also limited. Finally, the assessment of the relationship between spatial abilities enhancement and anatomy knowledge scores was qualitative. Consequently, a quantitative meta‐analysis was not possible. These particular limitations impact the findings of the study and implications for application to practice.

## 5 CONCLUSION

Enhanced spatial abilities seem to transfer mainly to similar tasks related to spatial learning of anatomy. However, the spatial abilities tests used to evaluate spatial abilities were all intrinsic × dynamic. A future study using different spatial abilities tests could evaluate if spatial abilities engaging different dimensions of spatial cognition could be related to different spatial anatomy tasks.

## AUTHOR CONTRIBUTIONS

**Lara Nokovitch:** Conceptualization; design; data acquisition; data analysis; writing—original draft. **Houssein El Hajj:** Data acquisition; data analysis. **Sophie Deneuve:** Writing—review and editing; final approval. **Vincent De Andrade:** Data acquisition. **Rémi Gagnayre:** Writing—review and editing; final approval. **Aurore Margat:** Writing—review and editing; final approval.

## CONFLICT OF INTEREST STATEMENT

The authors declare no conflicts of interest.

## ETHICAL APPROVAL

Authors declare human ethics approval was not needed for this study.

## Supporting information


**Appendix S1:** Research equations.

**Appendix S2:** Excluded articles.

**Appendix S3:** Quality evaluation of the included articles.

## Data Availability

The data that support the findings of this study are available from the corresponding author upon reasonable request.
