# Supplementary material for: Visual–spatial abilities enhancement and spatial anatomy learning: A systematic review
Source: Med Educ. 2025 Aug 19;59(12):1322–32. doi: 10.1111/medu.70022 (PMC12686770; doi:10.1111/medu.70022)
Supplement: Supplementary file 1 — Appendix S1: Research equations. [file MEDU-59-1322-s001.docx]

**Appendix 1 : Research equations.**

| **Pubmed** | ((((pedagog*[TW] OR education*[TW] OR "education"[MH] OR "literacy"[MH] OR literac*[TW] OR "health literacy"[MH] OR "computer literacy"[MH] OR "information literacy"[MH] OR "visual-spatial skills"[TW] OR "visual-spatial thinking"[TW] OR "visual-spatial abilities"[TW] OR "cognitive visualization"[TW])) AND ((student*[TW] OR "Students, Medical"[MH] OR "education, medical"[MH]))) AND (("anatomy"[MH] OR anatomy[TW] OR anatomies[TW]))) AND ((spatial*[TW] OR "3D"[TW] OR "4D"[TW])) |
| --- | --- |
| **Embase** | (pedagog*:ti,ab,kw OR education*:ti,ab,kw OR ‘education’/exp OR literac*:ti,ab,kw OR ‘literacy’/exp OR ‘health literacy’/exp OR ‘computer literacy'/exp OR 'information literacy'/exp OR 'visual-spatial skills':ti,ab,kw OR 'visual-spatial thinking':ti,ab,kw OR 'visual-spatial abilities':ti,ab,kw OR 'cognitive visualization':ti,ab,kw) AND (student*:ti,ab,kw OR 'medical student'/exp OR 'medical education'/exp) AND ('anatomy'/exp OR anatomy:ti,ab,kw OR anatomies:ti,ab,kw) AND (spatial*:ti,ab,kw OR '3D':ti,ab,kw OR '4D':ti,ab,kw) |
| **Scopus** | ( ( TITLE-ABS-KEY ( "spatial" )  OR  TITLE-ABS-KEY ( "3D" )  OR  TITLE-ABS-KEY ( "4D" ) ) )  AND  ( ( TITLE-ABS-KEY ( anatomy )  OR  TITLE-ABS-KEY ( anatomies ) ) )  AND  ( TITLE-ABS-KEY ( student* ) )  AND  ( ( TITLE-ABS-KEY ( pedagog* )  OR  TITLE-ABS-KEY ( education* )  OR  TITLE-ABS-KEY ( literacy )  OR  TITLE-ABS-KEY ( "health literacy" )  OR  TITLE-ABS-KEY ( "computer literacy" )  OR  TITLE-ABS-KEY ( "information literacy" )  OR  TITLE-ABS-KEY ( "visual spatial skills" )  OR  TITLE-ABS-KEY ( "visual spatial thinking" )  OR  TITLE-ABS-KEY ( "visual spatial abilities" )  OR  TITLE-ABS-KEY ( "cognitive visualization" ) ) ) |
| **ERIC** | (pedagog* OR education* OR literacy OR literac* OR "health literacy" OR "computer literacy" OR "information literacy" OR "visual-spatial skills" OR "visual spatial thinking" OR "visual spatial abilities" OR "cognitive visualization") AND (student*) AND ("anatomy" OR "anatomies") AND ("spatial" OR "3D" OR "4D") |
| **Web of Science** | **#1 AND #2 AND #3 AND #4**  [740](https://www-webofscience-com.ezproxy.u-paris.fr/wos/woscc/summary/2219c3ae-985d-4b3b-bf23-184665f25685-53c8357e/relevance/1)  **((ALL=(spatial*)) OR ALL=("3D")) OR ALL=("4D")**  [2,232,904](https://www-webofscience-com.ezproxy.u-paris.fr/wos/woscc/summary/e1b6c301-026d-4936-bde3-34f34c359373-53c82ea4/relevance/1)  **((TS=(anatomy)) OR ALL=(anatomy)) OR ALL=(anatomies)**  [701,138](https://www-webofscience-com.ezproxy.u-paris.fr/wos/woscc/summary/eb7b56e6-f2c9-43b8-bbf4-6b8ea08d7480-53c81acf/relevance/1)  **((ALL=(student*)) OR TS=("students, medical")) OR TS=("education, medical")**  [1,469,813](https://www-webofscience-com.ezproxy.u-paris.fr/wos/woscc/summary/69b54302-9b92-42e7-a04a-1acec50c6aaf-53c80774/relevance/1)  **(((((((((((ALL=(pedagog*)) OR ALL=(education*)) OR TS=(education)) OR TS=(literacy)) OR ALL=(literac*)) OR TS=("health literacy")) OR TS=("computer literacy")) OR TS=("information literacy")) OR ALL=("visual-spatial skills")) OR ALL=("visual-spatial thinking")) OR ALL=("visual-spatial abilities")) OR ALL=("cognitive visualization")** |
