# Supplementary material for: Visual–spatial abilities enhancement and spatial anatomy learning: A systematic review
Source: Med Educ. 2025 Aug 19;59(12):1322–32. doi: 10.1111/medu.70022 (PMC12686770; doi:10.1111/medu.70022)
Supplement: Supplementary file 2 — Appendix S2: Excluded articles. [file MEDU-59-1322-s002.docx]

**Appendix 2 :** Excluded articles.

| Spatial ability test only at baseline (n = 36) | 1. Allen LK, Eagleson R, de Ribaupierre S. Evaluation of an online three-dimensional interactive resource for undergraduate neuroanatomy education. Anat Sci Educ. oct 2016;9(5):431‑9. 2. Bogomolova K, Vorstenbosch MATM, El Messaoudi I, Holla M, Hovius SER, van der Hage JA, et al. Effect of binocular disparity on learning anatomy with stereoscopic augmented reality visualization: A double center randomized controlled trial. Anat Sci Educ. janv 2023;16(1):87‑98. 3. Bogomolova K, van der Ham IJM, Dankbaar MEW, van den Broek WW, Hovius SER, van der Hage JA, et al. The Effect of Stereoscopic Augmented Reality Visualization on Learning Anatomy and the Modifying Effect of Visual-Spatial Abilities: A Double-Center Randomized Controlled Trial. Anatomical Sciences Education. 2020;13(5):558‑67. 4. Bölek KA, De Jong G, Henssen D. The effectiveness of the use of augmented reality in anatomy education: a systematic review and meta-analysis. Sci Rep. 27 juill 2021;11(1):15292. 5. Bork F, Stratmann L, Enssle S, Eck U, Navab N, Waschke J, et al. The Benefits of an Augmented Reality Magic Mirror System for Integrated Radiology Teaching in Gross Anatomy. Anat Sci Educ. nov 2019;12(6):585‑98. 6. Brewer-Deluce D, Bak AB, Simms AJ, Sinha S, Mitchell JP, Shin D, et al. Virtual Reality Bell-Ringer: The Development and Testing of a Stereoscopic Application for Human Gross Anatomy. Anat Sci Educ. mai 2021;14(3):330‑41. 7. Brewer DN, Wilson TD, Eagleson R, de Ribaupierre S. Evaluation of neuroanatomical training using a 3D visual reality model. Stud Health Technol Inform. 2012;173:85‑91. 8. Cappellen van Walsum AM van, Henssen DJHA. E-Learning Three-Dimensional Anatomy of the Brainstem: Impact of Different Microscopy Techniques and Spatial Ability. Anat Sci Educ. 28 janv 2021; 9. Delisser PJ, Carwardine D. Student Perceptions of Sectional CT/MRI Use in Teaching Veterinary Anatomy and the Correlation with Visual Spatial Ability: A Student Survey and Mental Rotations Test. J Vet Med Educ. Fall 2018;45(3):320‑9. 10. Garg A, Norman G, Spero L, Taylor I. Learning anatomy: Do new computer models improve spatial understanding? Medical Teacher. 1999;21(5):519‑22. 11. Guillot A, Champely S, Batier C, Thiriet P, Collet C. Relationship between spatial abilities, mental rotation and functional anatomy learning. Adv Health Sci Educ Theory Pract. nov 2007;12(4):491‑507. 12. Ho S, Liu P, Palombo D, Handy T, Krebs C. The role of spatial ability in mixed reality learning with the HoloLens. ANATOMICAL SCIENCES EDUCATION. 13. Jang S, Vitale JM, Jyung RW, Black JB. Direct manipulation is better than passive viewing for learning anatomy in a three-dimensional virtual reality environment. Computers and Education. 2017;106:150‑65. 14. Keedy AW, Durack JC, Sandhu P, Chen EM, O’Sullivan PS, Breiman RS. Comparison of traditional methods with 3D computer models in the instruction of hepatobiliary anatomy. Anat Sci Educ. avr 2011;4(2):84‑91. 15. Knudsen L, Nawrotzki R, Schmiedl A, Mühlfeld C, Kruschinski C, Ochs M. Hands-on or no hands-on training in ultrasound imaging: A randomized trial to evaluate learning outcomes and speed of recall of topographic anatomy. Anatomical Sciences Education. 2018;11(6):575‑91. 16. Koh MY, Tan GJS, Mogali SR. Spatial ability and 3D model colour-coding affect anatomy performance: a cross-sectional and randomized trial. Sci Rep. 15 mai 2023;13(1):7879. 17. Kurul R, Ogun M, Narin A, Avci S, Yazgan B. An Alternative Method for Anatomy Training: Immersive Virtual Reality. ANATOMICAL SCIENCES EDUCATION. sept 2020;13(5):648‑56. 18. Labranche L, Wilson T, Terrell M, Kulesza R. Learning in Stereo: The Relationship Between Spatial Ability and 3D Digital Anatomy Models. ANATOMICAL SCIENCES EDUCATION. mars 2022;15(2):291‑303. 19. Langlois J, Hamstra SJ, Dagenais Y, Lemieux R, Lecourtois M, Yetisir E, et al. Objects drawn from haptic perception and vision-based spatial abilities. Anat Sci Educ. mars 2024;17(2):433‑43. 20. Lischka M, Gittler G. Spatial abilities and learning modes in anatomy beginners. In: Scherpbier A, VanderVleuten C, Rethans J, VanderSteeg A, éditeurs. 1997. p. 166‑9. 21. Martin JF, Linton A, Svenson GR, Garrett AC, Mango DW, Svec PM, et al. Landmark Positioning on a Map: An Alternative Measure of Spatial Ability for Identifying Students Who May Benefit From Learning Gross Anatomy with Virtual  Reality. J Vet Med Educ. 4 sept 2024;e20240011. 22. McBain KA, Habid R, Laggis G, Quaiattini A, Ventura N, Noel GPJC. Scoping Review: The Use of Augmented Reality in Clinical Anatomical Education and Its Assessment Tools. Anat Sci Educ [Internet]. 2021;((McBain K.A.; Laggis G.) School of Physical and Occupational Therapy, McGill University, Montreal, QC, Canada). 23. Meyer ER, James AM, Sinning A, Thompson K, Cui D. A Pilot Study of the Impact of Three-Dimensional Stereoscopic Models of Pelvic Anatomy on Short- and Long-Term Retention in First-Year Medical Students. HAPS Educator. 2020;24(3):7‑22. 24. Miller G, Scerbo M, Zybak S, Byars D, Goodmurphy C, Lattanzio F, et al. Learner Improvement From a Simulation-Enhanced Ultrasonography Curriculum for First-Year Medical Students. JOURNAL OF ULTRASOUND IN MEDICINE. mars 2017;36(3):609‑19. 25. Newman H, Carr S, Meyer A. Role of spatial ability, motivation and anxiety in learning neuroanatomy. FOCUS ON HEALTH PROFESSIONAL EDUCATION-A MULTIDISCIPLINARY JOURNAL. 2022;23(1). 26. Nguyen N, Mulla A, Nelson AJ, Wilson TD. Visuospatial anatomy comprehension: the role of spatial visualization ability and problem-solving strategies. Anat Sci Educ. 2014;7(4):280‑8. 27. Nguyen N, Nelson AJ, Wilson TD. Computer visualizations: factors that influence spatial anatomy comprehension. Anat Sci Educ. avr 2012;5(2):98‑108. 28. Nilsson TA, Hedman LR, Ahlqvist JB. A randomized trial of simulation-based versus conventional training of dental student skill at interpreting spatial information in radiographs. Simul Healthc. 2007;2(3):164‑9. 29. Petekkaya E, Ünalmış Aykar D, Kaptan Z. An analysis of the relationship of « the Mozart effect » with BDNF levels in anatomy education. Anat Sci Educ. juin 2024;17(4):770‑8. 30. Ritchie HE, Oakes D, Drury H, Ollerenshaw S, Hegedus E. Can drawing instruction help students with low visuospatial ability in learning anatomy? Anat Sci Educ. mars 2023;16(2):252‑65. 31. Schirone R, Corte GM, Ehlers JP, Herre C, Schmedding M, Merle R, et al. Effects of 3D Scans on Veterinary Students’ Learning Outcomes Compared to Traditional 2D Images in Anatomy Classes. Animals (Basel). 25 juill 2024;14(15). 32. Tan S, Hu A, Wilson T, Ladak H, Haase P, Fung K. Role of a computer-generated three-dimensional laryngeal model in anatomy teaching for advanced learners. J Laryngol Otol. avr 2012;126(4):395‑401. 33. Van Nuland SE, Rogers KA. The anatomy of E-Learning tools: Does software usability influence learning outcomes? Anat Sci Educ. 8 juill 2016;9(4):378‑90. 34. Van Nuland SE, Rogers KA. The skeletons in our closet: E-learning tools and what happens when one side does not fit all. Anat Sci Educ. nov 2017;10(6):570‑88. 35. Yao C, Chow J, Choi W, Mattheos N. Measuring the impact of simulation practice on the spatial representation ability of dentists by means of Impacted Mandibular Third Molar (IMTM) Surgery on 3D printed models. EUROPEAN JOURNAL OF DENTAL EDUCATION. août 2019;23(3):332‑43. 36. Yohannan DG, Oommen AM, Amogh BJ, Raju NK, Suresh RO, Nair SJ. « Air Anatomy » - Teaching Complex Spatial Anatomy Using Simple Hand Gestures. Anat Sci Educ. 15 avr 2021. |
| --- | --- |
| No spatial anatomy knowledge assessement (n = 9) | 1. Bogomolova K, Hierck BP, van der Hage JA, Hovius SER. Anatomy Dissection Course Improves the Initially Lower Levels of Visual-Spatial Abilities of Medical Undergraduates. Anatomical Sciences Education. 2020;13(3):333‑42. 2. Gonzales RA, Ferns G, Vorstenbosch MATM, Smith CF. Does spatial awareness training affect anatomy learning in medical students? Anat Sci Educ. nov 2020;13(6):707‑20. 3. Gutierrez JC, Holladay SD, Arzi B, Clarkson C, Larsen R, Srivastava S. Improvement of spatial and nonverbal general reasoning abilities in female veterinary medical students over the first 64 weeks of an integrated curriculum. Front Vet Sci [Internet]. 2019;6(MAY). 4. Gutierrez JC, Chigerwe M, Ilkiw JE, Youngblood P, Holladay SD, Srivastava S. Spatial and Visual Reasoning: Do These Abilities Improve in First-Year Veterinary Medical Students Exposed to an Integrated Curriculum? J Vet Med Educ. Winter 2017;44(4):669‑75. 5. Hoyek N, Collet C, Rastello O, Fargier P, Thiriet P, Guillot A. Enhancement of mental rotation abilities and its effect on anatomy learning. Teach Learn Med. juill 2009;21(3):201‑6. 6. Koh MY, Tan GJS, Mogali SR. Spatial ability and 3D model colour-coding affect anatomy performance: a cross-sectional and randomized trial. Sci Rep. 15 mai 2023;13(1):7879. 7. Langlois J, Wells GA, Lecourtois M, Bergeron G, Yetisir E, Martin M. Spatial abilities in an elective course of applied anatomy after a problem-based learning curriculum. Anat Sci Educ. juin 2009;2(3):107‑12. 8. Roach VA, Fraser GM, Kryklywy JH, Mitchell DGV, Wilson TD. Guiding Low Spatial Ability Individuals through Visual Cueing: The Dual Importance of Where and When to Look. Anat Sci Educ. janv 2019;12(1):32‑42. 9. Sezer B, Sezer TA, Elcin M. Exploring spatial ability in healthcare students and the relationship to training with virtual and actual objects. Eur J Dent Educ. 19 juin 2021; 10. Vorstenbosch MATM, Klaassen TPFM, Donders ARTR, Kooloos JGM, Bolhuis SM, Laan RFJM. Learning anatomy enhances spatial ability. Anat Sci Educ. août 2013;6(4):257‑62. |
| Relationships between spatial abilities and spatial anatomy scores unexplored (n = 3) | 1. Lufler RS, Zumwalt AC, Romney CA, Hoagland TM. Effect of visual-spatial ability on medical students’ performance in a gross anatomy course. Anat Sci Educ. févr 2012;5(1):3‑9. 2. McBain K, Chen L, Lee A, O’Brien J, Ventura NM, Noël GPJC. Evaluating the Integration of Body Donor Imaging into Anatomical Dissection Using Augmented Reality. Anat Sci Educ. 30 nov 2021; 3. Na Y, Clary DW, Rose-Reneau ZB, Segars L, Hanson A, Brauer P, et al. Spatial Visualization of Human Anatomy through Art Using Technical Drawing Exercises. Anat Sci Educ. 2 avr 2021; |
