# Supplementary material for: Visual–spatial abilities enhancement and spatial anatomy learning: A systematic review
Source: Med Educ. 2025 Aug 19;59(12):1322–32. doi: 10.1111/medu.70022 (PMC12686770; doi:10.1111/medu.70022)
Supplement: Supplementary file 3 — Appendix S3: Quality evaluation of the included articles. [file MEDU-59-1322-s003.docx]

# Appendix 3: Quality evaluation of the included articles according to the BEME criteria.

| **BEME Criterion** | **Guimaraes et al., 2018** | **Hontoir et al., 2022** | **Provo et al., 2002** | **Cui et al., 2017** | **Lufler et al., 2011** | **Harmon et al., 2022** | **Yousuf et al., 2023** | **Hegarty et al., 2009** |
| --- | --- | --- | --- | --- | --- | --- | --- | --- |
| **1. Research question: Is the research question or hypothesis clearly stated?** | Yes, the research question is clearly stated: evaluating the effect of computer assisted learning on spatial abilities. | Yes, the research question is clear: is online teaching of radiographic anatomy as effective as classroom-based teaching? | Yes, the research question is clearly stated: does studying a cross-section of a canine head improve 3D anatomical visualization? | Yes, the research question is clearly stated: are 3D stereoscopic models efficacious teaching tools for head and neck vascular anatomy compared to traditional methods? | Yes, the study clearly investigates whether visual-spatial abilities are related to anatomy performance. | Yes, the research question is clearly stated: evaluating the influence of an anatomy course for physical therapy students on visual-spatial abilities. | Yes, the research aim is clearly stated: evaluating the effect of different factors such as video learning of anatomy, training, gender and type of practical exam on mental rotation ability. | Yes, the research question is clearly defined: evaluating if dental education enhances an individual’s spatial competence. |
| **2. Study subjects: Is the subject group appropriate for the study being carried out?** | Yes, medical students. | Yes, second-year veterinary students. | Yes, first-year veterinary students. | Yes, first-year medical students. | Yes, first-year medical students. | Yes, first-year physical therapy students. | Yes, second and fifth-year medical students. | Yes, first-year dentistry students. |
| **3. Data collection methods: Are the methods used appropriate for the research question and context?** | Yes, methods (intervention group with pre-test and post-test design) are appropriate and well justified. | Yes, methods (randomized experiment) are appropriate and well justified. | Yes, methods (randomized experiment) are appropriate and well justified. | Yes, methods (intervention and control group with pre-test and post-test design) are appropriate and well justified. | Yes, methods (intervention group with only post-test design) are appropriate and well justified. | Yes, methods (intervention group only with post-test design) are appropriate and well justified. | Yes, methods (intervention and control group with pre-test and post-test design) are appropriate and well justified. | Yes, methods (intervention group only with post-test design) are appropriate and well justified. |
| **4. Completeness of data: Attrition rates/acceptable questionnaire response rates** | Yes, poor attrition rate (0%) and high completion rate (100%). | Yes, poor attrition rate (0%) and high completion rate (94%). | Yes, poor attrition rate (0%) and high completion rate (100%). | Yes, poor attrition rate (0%) and high completion rate (100%). | Yes, poor attrition rate (0%) and high completion rate (100%). | Yes, poor attrition rate (2%) and high completion rate (90%). | Yes, poor attrition rate (0%), and high completion rates (96% for MRT1 and 70% for MRT2). | Yes, poor attrition rate (0%) and high completion rate (100%). |
| **5. Risk of bias assessment: Is a statement of author positionality and a risk of bias assessment included?** | No explicit positionality or structured risk of bias assessment was provided. | No explicit positionality or structured risk of bias assessment was provided. | No explicit positionality or structured risk of bias assessment was provided. | No explicit positionality or structured risk of bias assessment was provided. | No explicit positionality or structured risk of bias assessment was provided. | No explicit positionality or structured risk of bias assessment was provided. | No explicit positionality or structured risk of bias assessment was provided. | No explicit positionality or structured risk of bias assessment was provided. |
| **6. Analysis of results: Are the statistical and other methods of results analysis used appropriate?** | Yes, statistical methods including t-tests, correlations, and regressions were appropriate and well applied. | Yes, statistical methods including t-tests, correlations, and a Kruskal-Wallis test were appropriate and well applied. | Yes, statistical methods including t-tests and correlations were appropriate and clearly explained. | Yes, statistical methods included appropriate statistical tests (t-tests, chi-square, etc.) and were clearly explained. | Yes, statistical methods including t-tests, correlations and covariance analysis were appropriate and clearly explained. | Yes, appropriate statistical methods (t-tests, analysis of covariance, correlations) were used and well explained. | Yes, appropriate statistical methods (t-tests, correlations) were used and well explained. | Yes, statistical analyses (t-tests, correlations, etc) were appropriate but not well justified in the methods section. |
| **7. Conclusions: Is it clear that the data justify the conclusions drawn?** | Yes, conclusions are well supported by the data with a dose-dependent effect of computer assisted learning training in anatomy on spatial abilities. | Yes, conclusions align with the data showing no differences between mental rotation and radio-anatomy scores between groups at post-test. | Yes, conclusions are well supported by the data with positive correlations between spatial abilities scores and drawing of views. | Yes, conclusions are well supported by the data, students exposed to the 3D stereoscopic models increasing their ability to correctly identify anatomical structures. | Yes, conclusions are well supported by the data with positive trend between spatial abilities test scores and practical examination. | Yes, conclusions align with the data showing improved mental rotation test scores following the anatomy course. | Yes, conclusions align with the data although no statistically significant correlation between mental rotation scores and anatomy scores was found. | Yes, the findings support the conclusion that spatial ability relates to restorative dentistry and improves for domain-specific tasks. |
| **8. Reproducibility: Could the study be repeated by other researchers?** | Yes, detailed methods, sample description, and tools allow reproducibility. | Yes, methods are sufficiently described to allow repetition by others. | Yes, experimental procedures and instruments are sufficiently described to allow replication. | Yes, study design, procedures, and tools are described in sufficient detail to enable replication. | Yes, methods are clearly described allowing reproducibility of the study. | Yes, the study provides sufficient methodological detail for replication. | Yes, methods and tools are clearly described and replicable by other researchers. | Yes, sufficient methodological detail allows for reproducibility. |
| **9. Prospectivity: Is the study prospective?** | Yes, prospective design. | Yes, prospective design. | Yes, prospective design. | Yes, prospective design. | Yes, prospective design. | Yes, prospective design. | Yes, prospective design. | Yes, prospective design. |
| **10. Ethical issues: Are all ethical issues articulated and managed appropriately?** | Yes, the research was approved by the ethical committee, and written informed consent was obtained from all participants. | Yes, the research was reviewed by the ethical committee and followed the General Data Protection regulation. | Yes, ethical considerations were clearly stated, including voluntary participation and consent. | Yes, ethical approval and informed consent are explicitly mentioned. | Yes, the research was reviewed by the ethical committee, and participation in the study was voluntary. | Yes, the research was reviewed by the ethical committee. | Yes, the research was approved by the ethical committee, and recruited participants were given a consent form. | No, although participant consent is required, there is no mention of a review of the research by the ethical committee. |
| **11. Triangulation: Were results supported by data from more than one source?** | Partial: relies mostly on MRT scores, consistency in findings among groups. | Partial: relies mostly on MRT scores and radiographic anatomy test scores ; some degree of triangulation. | Partial: data was mainly collected from written tests and exams but included different instruments (head test, live-dog exam). | Partial: MRT scores and anatomy test scores are the main source of data, though some survey-based triangulation is provided. | Partial: results are based on MRT scores and anatomy tests scores, which are both performance measures, but not from multiple data sources. | Partial: the analysis includes multiple predictors, MRT scores and anatomy tests scores but relies mostly on quantitative data. | Yes, multiple data types were used to evaluate the effect of video learning of anatomy, ensuring triangulation. | Partial: multiple data types were used, but all evaluated student self-performance. |

MRT: Mental Rotation Test
